# Identification of Inhibitors of the *Schistosoma mansoni* VKR2 Kinase Domain

**DOI:** 10.1021/acsmedchemlett.2c00248

**Published:** 2022-10-05

**Authors:** Indran Mathavan, Lawrence J. Liu, Sean W. Robinson, Nelly El-Sakkary, Adam Jo J. Elatico, Darwin Gomez, Ricky Nellas, Raymond J. Owens, William Zuercher, Iva Navratilova, Conor R. Caffrey, Konstantinos Beis

**Affiliations:** † Department of Life Sciences, Imperial College London, Exhibition Road, London, South Kensington SW7 2AZ, United Kingdom; ‡ Rutherford Appleton Laboratory, Research Complex at Harwell, Didcot, Oxfordshire OX11 0FA, United Kingdom; § Center for Discovery and Innovation in Parasitic Diseases, Skaggs School of Pharmacy and Pharmaceutical Sciences, University of California San Diego, 9500 Gilman Drive, La Jolla, California 92093, United States; ∥ Kinetic Discovery Ltd., an Exscientia group company, The Schrödinger Building, Oxford Science Park, Oxford OX4 4GE, United Kingdom; ⊥ Institute of Chemistry, College of Science, University of the Philippines Diliman, Quezon City, Philippines 1101; # The Rosalind Franklin Institute, Harwell Campus, Didcot, OX11 0QX, United Kingdom; ∇ Division of Structural Biology, The Wellcome Centre for Human Genetics, University of Oxford, Oxford OX3 7BN, United Kingdom; ○ Structural Genomics Consortium, Division of Chemical Biology and Medicinal Chemistry, UNC Eshelman School of Pharmacy, Chapel Hill, North Carolina 27599, United States

**Keywords:** kinase domain, drug discovery, inhibitor, *Schistosoma*, schistosomiasis, crystal structure, docking, inhibition of autophosphorylation

## Abstract

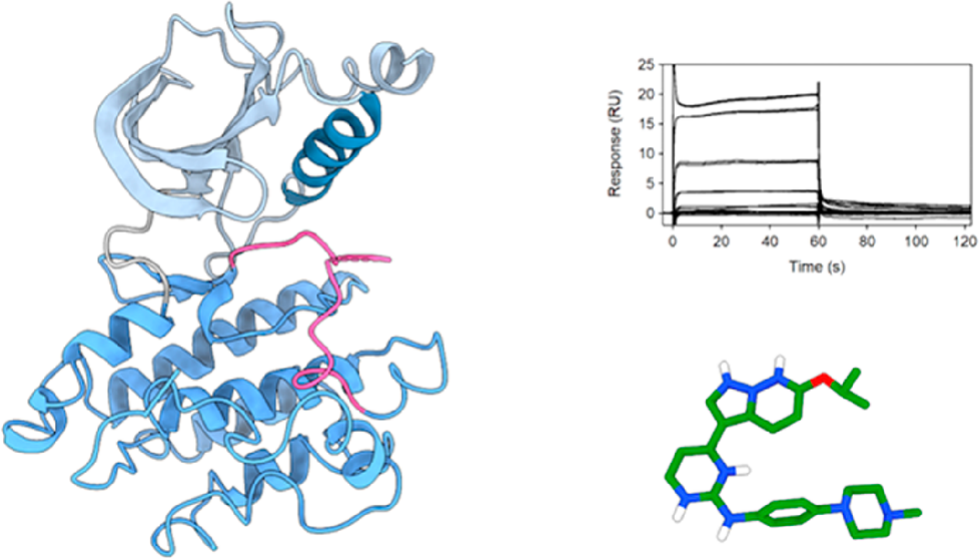

Schistosomiasis is a neglected tropical disease caused by parasitic flatworms. Current treatment relies on just one partially effective drug, praziquantel (PZQ). *Schistosoma mansoni* Venus Kinase Receptors 1 and 2 (SmVKR1 and SmVKR2) are important for parasite growth and egg production, and are potential targets for combating schistosomiasis. VKRs consist of an extracellular Venus Flytrap Module (VFTM) linked via a transmembrane helix to a kinase domain. Here, we initiated a drug discovery effort to inhibit the activity of the SmVKR2 kinase domain (SmVKR2_KD_) by screening the GSK published kinase inhibitor set 2 (PKIS2). We identified several inhibitors, of which four were able to inhibit its enzymatic activity and induced phenotypic changes in *ex vivoS. mansoni*. Our crystal structure of the SmVKR2_KD_ displays an active-like state that sheds light on the activation process of VKRs. Our data provide a basis for the further exploration of SmVKR2 as a possible drug target.

Hundreds of millions of people worldwide suffer from the parasitic disease known as schistosomiasis, which is caused by a trematode blood fluke of the genus *Schistosoma*.^1,2^ The three most medically important species are *Schistosoma hematobium*, *Schistosoma mansoni*, and *Schistosoma japonicum*. *S. hematobium* is the most common species with a presence in 54 countries, particularly in Africa.^3^*S. mansoni* is endemic in sub-Saharan Africa, Brazil, the Caribbean islands, Puerto Rico, Suriname, and Venezuela.^3^ Finally, *S. japonicum* is endemic in parts of the People’s Republic of China and the Philippines.^3^ The eggs of the parasite induce an inflammatory response that then leads to tissue fibrosis and portal vein hypertension or occlusion (intestinal schistosomiasis caused by *S. mansoni* and *S. japonicum*) or hydronephrosis and squamous bladder cancer (urinary schistosomiasis caused by *S. hematobium*).^4,1,5^ The greatest infection intensities are among children and adolescents, and, if left untreated, this painful and debilitating disease impairs academic performance and undermines social and economic development.^6−8^ Of note, female genital schistosomiasis has been linked to an increased risk of HIV infections^9,10^ and is now a major focus of World Health Organization (WHO) awareness campaigns.^11^

The current strategy to treat and control schistosomiasis focuses on decreasing morbidity through periodic treatment with the drug, praziquantel (PZQ), which is an acylated quinoline-pyrazine derivative.^12,13^ The WHO estimated that 236.6 million people required treatment for schistosomiasis in 2019 (https://www.who.int/news-room/fact-sheets/detail/schistosomiasis). PZQ acts on a calcium-permeable ion channel that is a member of the transient receptor potential melastatin channel subfamily.^14,15^ The drug causes rapid paralysis of the adult schistosome and damage to the worm’s surface (tegument).^12^ Although stable clinical resistance to the drug has yet to be reported, concern remains regarding the reliance on just one drug to treat whole populations of people. Further, the drug has a number of pharmaceutical and pharmacological drawbacks that encourage the search for new drugs.^13,16^

In recent years, the discovery of Venus Kinase Receptors (VKRs) in *S. mansoni* has offered new directions for schistosomiasis research drug discovery.^17,18^ VKR proteins are composed of a unique extracellular domain similar to class C G-protein coupled receptors that adopt a Venus Flytrap Module (VFTM).^18,17^ The VFTM of VKRs is connected to an intracellular tyrosine kinase domain via a single transmembrane helix. VKR proteins form homo- and heterodimers, a key requirement for VKR activation.^19^ VKR proteins are important in schistosome growth and egg production.^17^ Two VKR proteins, SmVKR1 and SmVKR2, have been cloned and characterized in *S. mansoni*, with l-arginine and calcium ions as the respective putative ligands.^20^ SmVKR1 activates the c-Jun N-terminal kinase (JNK) signal transduction pathway as determined by yeast two-hybrid screening.^17^ The JNK pathway is involved in oogenesis and the resumption of meiosis in *Caenorhabditis elegans*(21) and *Drosophila melanogaster*.^22^

VKRs are unique to invertebrates, making them attractive candidates for small molecule inhibition. To date, no VKR structure has been elucidated. The intracellular kinase domain of VKRs shares 41% sequence identity with that of insulin receptors (IRs).^23^ A known IR inhibitor, tyrphostin AG1024, inhibited both SmVKR1 and SmVKR2 and caused concentration-dependent apoptosis and cessation of egg production in schistosomes.^24^ The dual action of AG1024 on IR and VKR kinase domains is due to their conserved sequences and, possibly, structures.^24^

Here, we focused on small molecule discovery for the SmVKR2 kinase domain (SmVKR2_KD_). Specifically, we screened the GlaxoSmithKline (GSK) published kinase inhibitor set 2 (PKIS2; 645 molecules)^25^ against SmVKR2_KD_ recombinantly expressed in Sf9 insect cells and identified several low micromolar inhibitors. These were then screened against *ex vivo* adult *S. mansoni* for phenotypic changes; one inhibitor was markedly bioactive and a further three less so. The inhibitors inhibit the autophosphorylation activity of SmVKR2_KD_. We also determined the crystal structure of the SmVKR2_KD_ in complex with ADP, and based on the conformation of conserved kinase motifs, the SmVKR2_KD_ is in an active-like dimer state. The structure was used for *in silico* docking to predict the binding pose of the bioactive compounds.

Although both SmVKR1 and SmVKR2 are important for schistosome growth and egg production,^17^ we targeted SmVKR2 in our drug discovery approach as its expression is higher than that of SmVKR1.^20^ Because the kinase domain of the VKRs is linked to an extracellular domain by a transmembrane helix, several constructs varying in length were generated for expression in Sf9 insect cells. Specifically, 12 constructs with N- and C-terminal truncations were designed based on the sequence alignment of VKR1 and VKR2 from *S. hematobium* and *S. mansoni* using the Phyre2 and Pfam servers to try to maintain conserved kinase motifs (Figure S1). Small scale expression and purification experiments identified a SmVKR2_KD_ construct (residues 967–1308) suitable for further studies based on milligram expression levels as judged by Western blot. The protein displayed a monodisperse profile by size exclusion chromatography (Figure S2). After purification, the protein did not appear to be post-translationally modified or autophosphorylated as revealed by mass spectrometry analysis (Figure S2).

We employed surface plasmon resonance (SPR) to screen the PKIS2 library against the SmVKR2_KD_. The PKIS2 library comprises 645 small molecule inhibitors representing 86 diverse chemotypes.^25^ Twelve showed the strongest binding to the SmVKR2_KD_ with affinities between 0.57 and 170 μM (Table 1 and Figure S3). The identified inhibitors display a similar backbone consisting of either a 1H-pyrrolo[2,3-*b*]pyridine linked to benzene or a pyrimidine linked to benzene or pyrazolo[1,5-*b*]pyridazine (Table 1). GSK1520489 and GSK986310 had the highest affinities of 0.44 μM and 1.96 μM, respectively. Both compounds contain a 2,4-diaminopyrimidine as the putative hinge-binding moiety that likely drives binding.

**Table 1:**
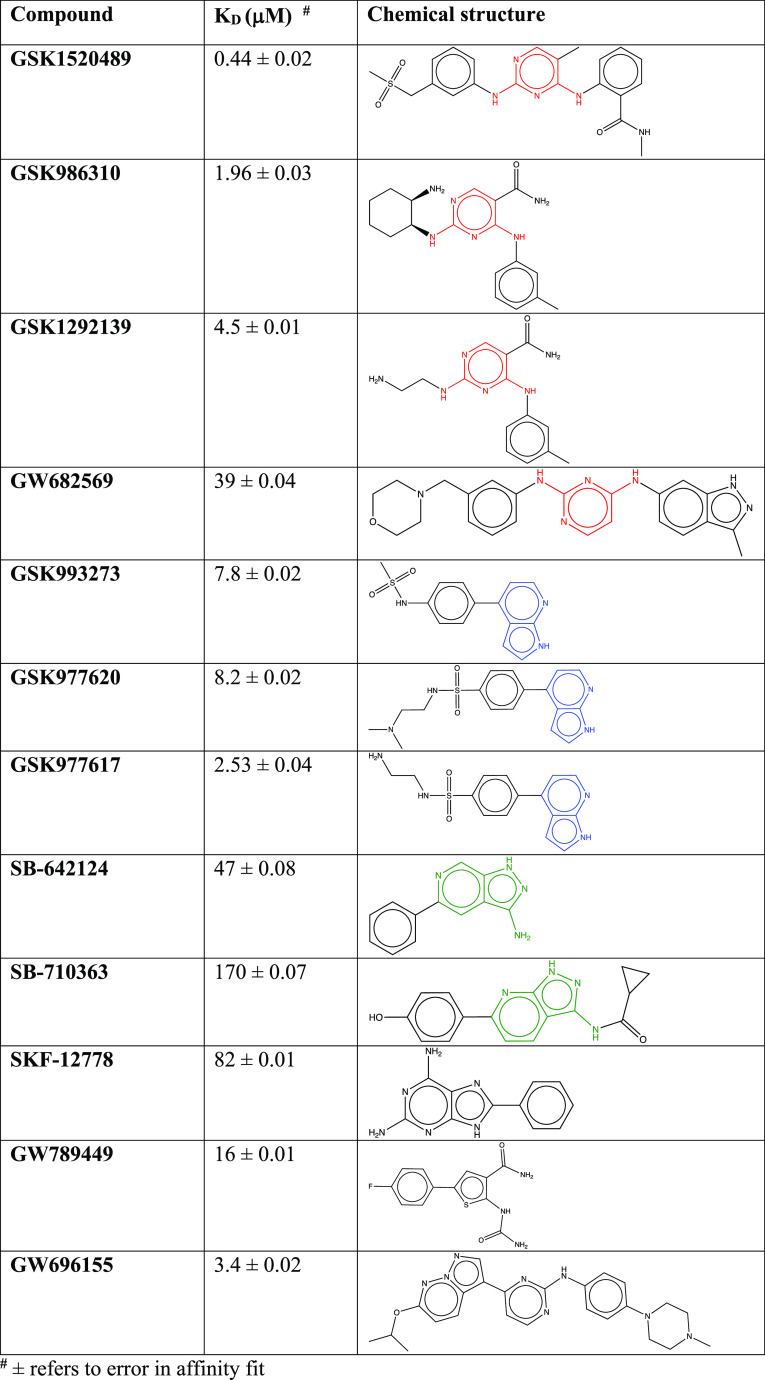
Small Molecule Inhibitors of the SmVKR2_KD_^a^

a Compounds are grouped by common compound substructure; red, 2,4-diaminopyrimidine; green, 7-azaindole; blue, 3-aminoindazole. Compounds in black do not have a common substructure.

The 12 compounds identified from the SPR screen were tested against *ex vivo* adult *S. mansoni* worms to investigate whether they induce phenotypic alterations in the parasite. Compound effects at 10 μM were assessed at 2, 5, and 24 h, and activity was partially quantified using an observation-based severity scoring system that is designed to holistically assess the many different responses to chemical insult of which the schistosome is capable.^26,27^ For those compounds eliciting phenotypic changes, WormAssay was also employed as an additional readout to measure average worm motility per well.^28,29^ Four compounds, GW696155, GSK986310, GSK1520489, and SB-710363, produced a variety of effects in the worm (Figure 1; Table 2; Table S1). GW696155 generated the strongest responses. By 2 h, the worms had decreased motility and lost their ability to adhere to the floor of the well (severity score of 2; Table 2). By 24 h, additional responses included worm degeneration and damage to the surface tegument in the form of blebs or bubbles (severity score of 4). These time-dependent observations were consistent with a decrease in average worm motility over time as measured by WormAssay (Figure 1). SB-710363 and GSK986310 had a milder effect in decreasing worm movement (severity score of 1), again registered by WormAssay. GSK1520489 caused a mild uncoordinated motility in the worm as assessed visually without a long-term decrease in average motility as measured by WormAssay. Although, SPR identified 12 compounds against the SmVKR2_KD_ (Table 1), the absence of phenotypic activity for eight of these compounds (Table S2) could be due to their poor uptake by the parasite. It is also possible that the phenotypic changes noted for the four active compounds are only partially related or unrelated to engagement of the SmVKR2 target.

**Table 2:**
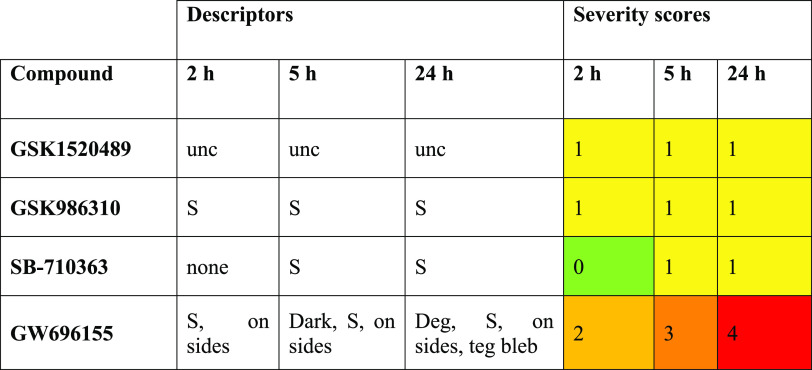
Phenotypic Changes of *S. mansoni* As a Function of Time Expressed As Descriptors and Severity Scores^a^

a Descriptor terms: degenerating (deg); uncoordinated (unc); slow (S); on sides, inability of worms to adhere to well floor with either the oral or ventral sucker; teg bleb, damage to the surface tegument. Representative data from three to five biological singleton assays with 10 μM compound.

**Figure 1:**
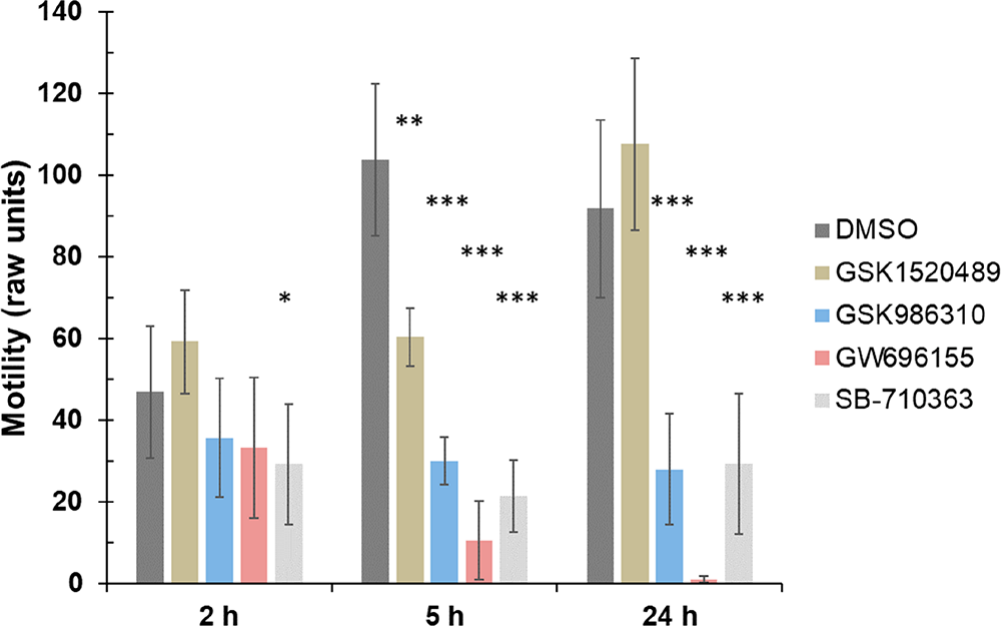
Changes in the average motility of adult *S. mansoni* as a function of time, as measured by WormAssay. Significance was determined by Student’s *t*-test (two-tailed): *P < 0.05, **p < 0.005, ***P < 0.0005. Data were derived from three to five biological assays with 10 μM compound.

To understand whether the bioactive compounds can inhibit the SmVKR2_KD_, we measured their IC_50_ values in the presence of 10 μM ATP; SB-710363 was not included in the measurements due to its weak *ex vivo* activity. In the absence of a known substrate for SmVKR2_KD_, we measured the ability of the compounds to inhibit the autophosphorylation capability of SmVKR2_KD_. GSK1520489 and GSK986310 inhibited activity with IC_50_ values of 6.47 μM and 5.69 μM, respectively, whereas GW696155 was less potent with a value of 20.15 μM, which could be attributed to its low aqueous solubility (a theoretical logP of 2.76; Figure 2). All three compounds inhibit the autophosphorylation activity of SmVKR2_KD_ and likely display competitive inhibition as the PKIS2 compounds bind to the ATP site of kinases.^25^

**Figure 2:**
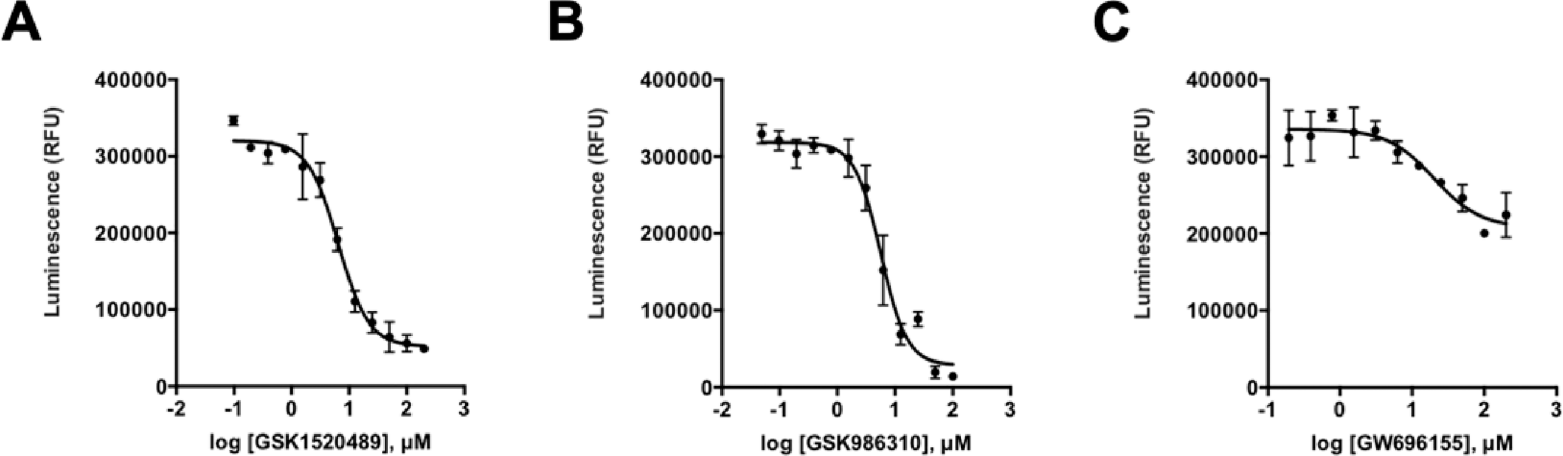
Compounds that are active against the parasite inhibit the autophosphorylation activity of SmVKR2_KD_. Concentration–response curves were developed for (A) GSK1520489, (B) GSK986310, and (C) GW696155. The inhibition measured for GW696155 is less than that of the other compounds, possibly due to its low solubility in the assay buffers. Assays were performed in triplicate, and error bars portray the standard deviations around the mean.

To support our drug discovery approach, we determined for the first time a crystal structure of the SmVKR2_KD_ at 3.0 Å resolution in the presence of ADP. SmVKR2 adopts a canonical bilobal kinase fold with an ADP molecule bound in the cleft between the two lobes (Figure 3). The N- and C-terminal lobes are formed mainly by β-sheets and α-helices, respectively, and both lobes are connected by a hinge. A novel feature of the SmVKR2_KD_ is the presence of an extended helix at the N-terminal lobe, which we termed α0. This helix probably extends toward the membrane as part of the transmembrane helix that links the kinase domain with the VFTM module. The SmVKR2_KD_ was crystallized as a dimer with the interface being stabilized by interactions between the N-lobe from one protomer and the C-lobe of the opposite protomer, related by a 2-fold symmetry. Although the crystals were grown in the presence of ATP-γ-S, the electron density maps only corresponded to the ADP moiety and not the thiophosphate group. Despite ATP-γ-S being a nonhydrolyzable ATP analogue, it can be slowly hydrolyzed at a rate 0.5% of that of ATP.^30^ As the crystals took over one month to grow, we believe that the ATP-γ-S was slowly hydrolyzed during crystallization and that we captured the posthydrolysis state of the kinase. In the electron density maps, we also observe weak density near the ADP and the catalytic D1118 that could correspond to the cleaved thiophosphate group (Figure S4): the distance between the ADP and this density is too far to correspond to a magnesium cation.

**Figure 3:**
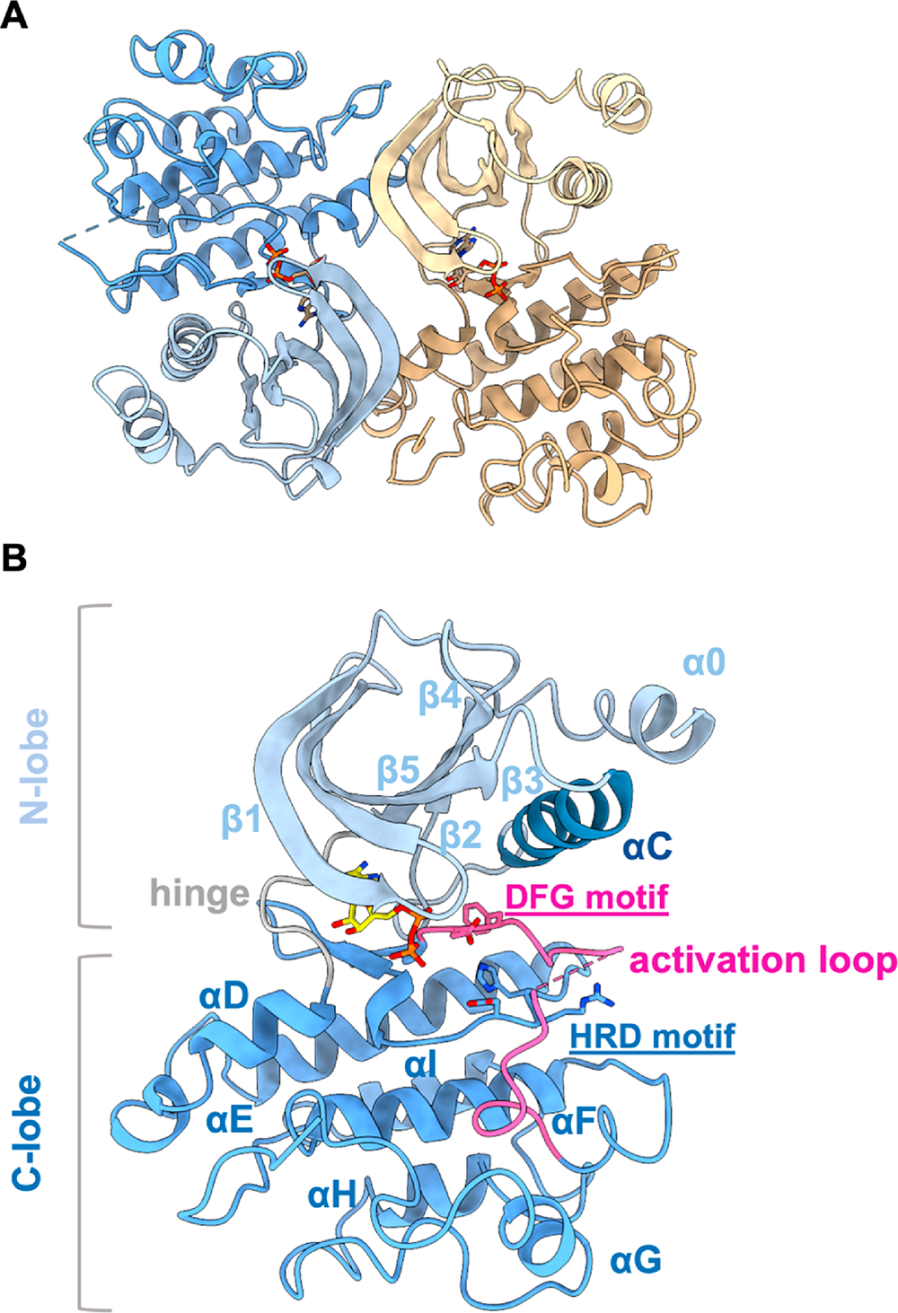
Crystal structure of the SmVKR2_KD_. (A) The SmVKR2_KD_ adopts a canonical kinase domain fold. In the presence of ADP, from ATP-γ-S hydrolysis, the structure has adopted a dimeric architecture stabilized by interactions between the N- and C-terminal lobes of the opposite monomers. Each monomer is shown as a cartoon with the N- and C-terminal lobes in light and dark colors, respectively. ADP is shown as sticks. (B) The SmVKR2_KD_ has adopted an active-like conformation based on the orientation of key motifs, including the αC-in and DFG-in conformations. Key motifs have been labeled. Same color scheme as panel A.

Based on the conformation and orientation of the hydrophobic spine, the αC-in DFG-in conformation,^31^ and the E1014 and D1118 pointing toward the ATP-binding site, the SmVKR2_KD_ has adopted an active-like conformation. The hydrophobic spine comprises the catalytic and regulatory spines. The complete catalytic spine or C-spine forms upon ATP binding (in our ADP-bound structure, the adenine ring of ADP brings the two lobes together) and consists of hydrophobic residues from both lobes, including A995 from the VAVK motif, and L1107 from the β7-strand, which sandwiches the ADP adenine ring. The regulatory spine or R-spine consists of L1029 from the αC β4 loop, M1018 from the αC helix, F1119 from the DFG motif, and H1098 from the HRD motif. Another feature of active kinases is the phosphorylation and conformation of the activation loop that is tightly associated with the C-lobe. In the SmVKR2_KD_, the loop is confined within the C-lobe reminiscent of active kinases, whereas in inactive kinases, it extends toward the N-lobe.^31^ Although the entire activation loop could be traced, we decided to partially model it as the density between residues 1124 and 1141 is too weak/disordered to confidently add side chains. The interaction of K997 and E1014, although weak at a distance of 3.8 Å, resembles the salt-bridge seen in active kinases that anchor the αC helix to the β3 strand.^31^ Overall, based on apparent similarities between the key structural features of the SmVKR2_KD_ and active kinases, we propose that the SmVKR2_KD_ structure represents an active-like state. Further, because the structure is in the presence of ADP, it most likely resembles the posthydrolysis state, and even though αC appears to be in the in-conformation, it shows a small degree of displacement toward the out-conformation relative to fully active kinases.^31,32^

Our efforts to capture the SmVKR2_KD_ in complex with the identified inhibitors yielded weakly diffracting crystals that were not suitable for further analysis. In an attempt to identify the binding pose of the inhibitors, we performed *in silico* docking using AutoDock Vina.^33^ As the PKIS2 compounds are ATP-competitive kinase inhibitors,^25^ we focused the search area within the ATP binding site. Although the resolution is limited to 3.0 Å and the density for water molecules or the magnesium cation was not observed, docking can still provide insights into the possible binding pose and interaction of the inhibitors within the ATP binding site. To evaluate our setup, we docked ATP and ADP and compared the latter to our crystal structure: the pose of the docked ADP displayed minor deviations from the crystal structure but within the acceptable limitations of the low-resolution structure (Figure S5; Table 3). The four compounds GSK1520489, GSK986310, GW696155, and SB-710363 that showed antischistosomal activity, of which the first three were also tested for and found to inhibit the enzymatic activity of the SmVKR2_KD_ were selected for docking, and their *in silico* affinities and binding poses measured (Table 3 and Figure 4, respectively). GSK1520489 is coordinated by interactions between its sulfone and amide groups with the side and main chains of D1051, respectively, and the amine (between the pyrimidine and sulfone group) within the main chain of R1104. The *N*-methylbenzamide of GSK1520489 is positioned within the hydrophobic spine of the ATP binding site and mimics the binding of the adenine ring of ADP. Binding of GSK986310 is mediated between the compound’s cyclohexylamine and the backbone carboxylate of R1104, and its amide and the side chain of either D1051 or S1054. The inhibitor’s *meta*-tolyl substituent is placed within the hydrophobic spine. GW696155, which displayed the greatest *ex vivo* activity, displays binding pose interactions between the pyrazolo[1,5-*b*]pyridazine ring and the side chain of K997 and the pyrimidine ring with the side chain of D1051. The piperazine ring is pointing toward the hydrophobic spine. Binding by SB-710363 is stabilized by interactions between its phenol ring and the main chain of M1047, located at the hinge, and by its cyclopropane carboxamide and the side chain of D1118 in the DFG motif.

**Table 3:** Binding Affinities for the Docked Nucleotides and Inhibitors As Calculated by Autodock Vina

compound name	affinity (kcal/mol)
ATP	–6.6
ATPγS	–7.6
ADP	–5.9
GSK1520489	–7.9
GSK986310	–7.7
GW696155	–8.0
SB-710363	–7.6

**Figure 4:**
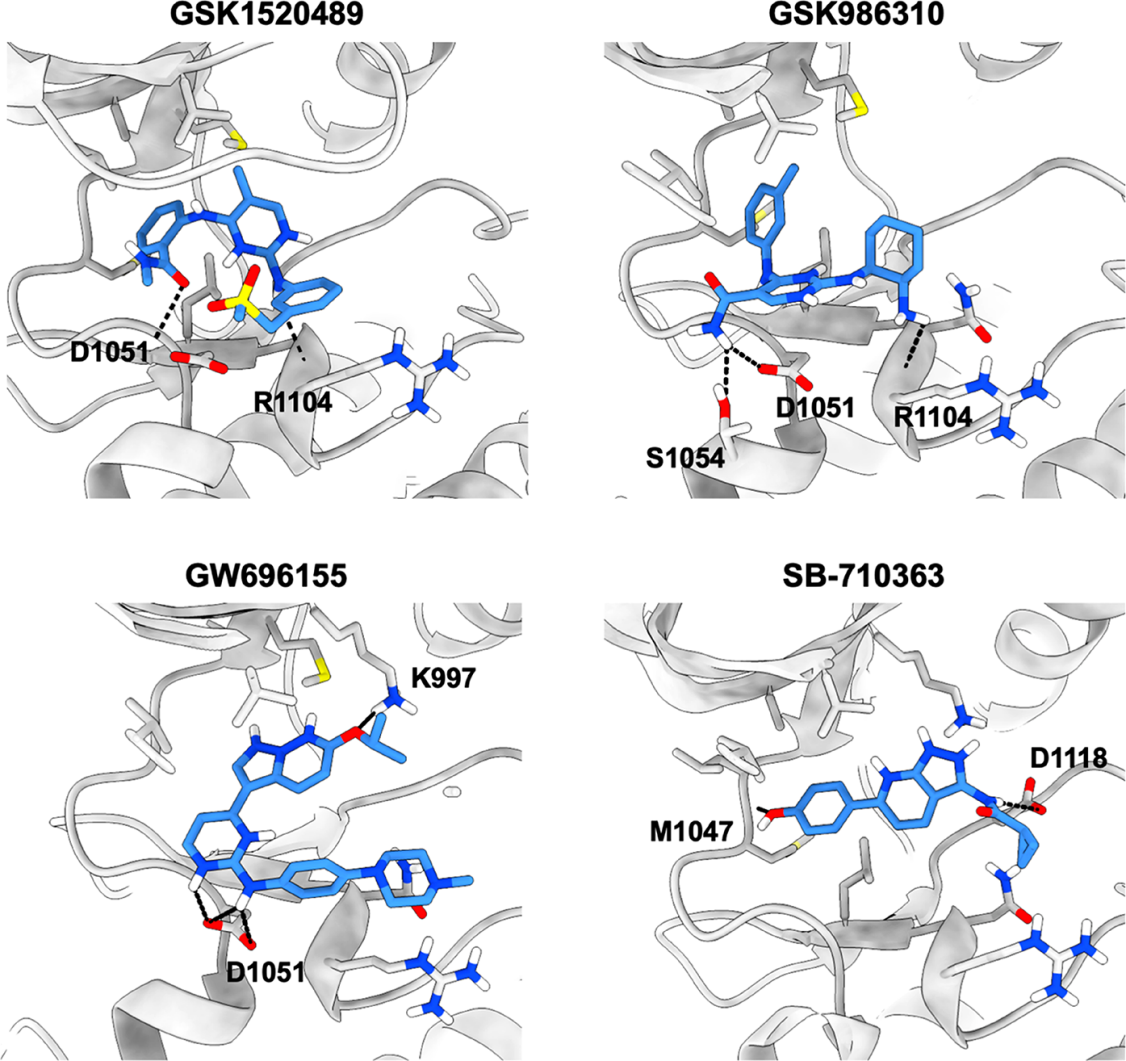
*In silico* docking poses of the four antischistosomal inhibitors in the SmVKR2_KD_ active site. The docked inhibitors display polar and hydrophobic interactions within the ATP binding site. The SmVKR2 kinase domain is shown as a gray cartoon and the interacting side chains and inhibitors as sticks. The carbon atoms of SmVKR2 and the inhibitor are colored in gray and light blue, respectively, and nitrogen, oxygen, and sulfur are colored dark blue, red, and yellow, respectively. Polar contacts are shown as black dashed lines.

Because treatment and control of schistosomiasis rely on just one partially effective drug, there is a need to identify alternative therapies. As a response to this insecure situation, we examined inhibitors of VKR as starting points for a new chemotherapy and, for the first time, identify inhibitors of the SmVKR2 receptor by targeting its kinase domain. Using the well characterized and freely available PKIS2 small molecule library from GSK,^25^ we identified a set of 12 compounds that displayed low micromolar SPR binding *in vitro* (Table 1), four of which, GSK1520489, GSK986310, GW696155, and SB-710363, were also active against *ex vivoS. mansoni* (Table 2). GSK1520489, GSK986310, and GW696155 inhibited the autophosphorylation activity of SmVKR2_KD_, and for the first two compounds, the IC_50_ values generated were similar to those derived from the SPR experiments.

Although the four compounds do not display significant chemical similarity based on the Tanimoto coefficient (despite the presence of either a pyrimidine or a pyrazolo[1,5-*b*]pyridazine group; Table S3), they can, nonetheless, efficiently bind and compete with ATP in the binding site. The compound with the strongest *ex vivo* antischistosomal activity was GW696155, which structurally resembles ATP, even though the Tanimoto coefficient is only 0.1. From our *in silico* docking studies, the pyrazolo[1,5-*b*]pyridazine and pyrimidine moieties of GW696155 are placed in a similar pose to the adenine and ribose moieties of ATP, respectively. GW696155 has been identified as an inhibitor of several human and parasite kinases^25,34^ and provides us with a useful starting point to explore analogues for improved potency and bioactivity against the schistosome parasite. The strong *ex vivo* activity of GW696155 could be attributed to its high membrane permeability but also to its nonselective nature^25,34^ by inhibiting other *S. mansoni* kinases.

Finally, our structural studies provide possible insights into how VKRs are activated. It has been suggested that VKRs need to dimerize upon ligand binding by the extracellular VFTM module that could then lead to dimerization of the kinase domain, autophosphorylation, and activation.^19^ The SmVKR2_KD_ structure is in an active-like conformation due to the orientation of key motifs that could resemble the ATP bound state after the VFTM module has dimerized.^19^ This is consistent with the recent structures of the full-length IR receptor.^35^ In our structure, we have also resolved a new feature for tyrosine kinases, namely, helix a0 (Figure 3). In the full length VKR, this helix would extend toward the membrane to form part of the transmembrane helix that links the kinase domain with the VFTM module.

In conclusion, we have identified initial lead inhibitors against the SmVKR2_KD_ that could pave the way to more potent inhibitors against the VKR2 receptor. Further, our resolved structure of the SmVKR2_KD_ will aid drug discovery efforts using *in silico* methods.

## Supporting Information Available

Materials and Methods, SPR data analysis, electron density maps, table with Tanimoto coefficient analysis of the inhibitors, crystallographic data collection, and refinement statistics table (PDF)

## Accession Codes

The coordinates and structure factors of the SmVKR2_KD_ have been deposited into the Protein Data Bank with the PDB ID code 7ZVS.
