# Supplementary material for: Identification of Inhibitors of the Schistosoma mansoni VKR2 Kinase Domain
Source: ACS Med Chem Lett. 2022 Oct 5;13(11):1715–22. doi: 10.1021/acsmedchemlett.2c00248 (PMC9661718; doi:10.1021/acsmedchemlett.2c00248)
Supplement: Supplementary file 1 — ml2c00248_si_001.pdf [file ml2c00248_si_001.pdf]

Identification of inhibitors of the *Schistosoma mansoni* VKR2 kinase domain

Indran Mathavan[1, 2, †], Lawrence J. Liu[3, †], Sean W. Robinson[4], Nelly El-Sakkary[3], Adam Jo J. Elatico[5, #], Darwin Gomez[5, ##], Ricky Nellas[5], Ray J. Owens[6, 7], William Zuercher[8, 9], Iva Navratilova[4], Conor R. Caffrey[3, *], Konstantinos Beis[1, 2, *]

[1] Department of Life Sciences, Imperial College London; Exhibition Road, London, South Kensington, SW7 2AZ, UK.

[2] Rutherford Appleton Laboratory, Research Complex at Harwell; Didcot, Oxfordshire OX11 0FA, UK.

[3] Center for Discovery and Innovation in Parasitic Diseases, Skaggs School of Pharmacy and Pharmaceutical Sciences, University of California San Diego, 9500 Gilman Drive, La Jolla, CA 92093, USA

[4] Kinetic Discovery Ltd, an Exscientia group company, The Schrödinger Building, Oxford Science Park, Oxford, OX4 4GE, United Kingdom

[5] Institute of Chemistry, College of Science, University of the Philippines Diliman, Quezon City Philippines 1101

[6] The Rosalind Franklin Institute, Harwell Campus, Didcot, OX11 0QX, UK.

[7] Division of Structural Biology, The Wellcome Centre for Human Genetics, University of Oxford, OX3 7BN, UK

[8] Structural Genomics Consortium, Division of Chemical Biology and Medicinal Chemistry, UNC Eshelman School of Pharmacy, Chapel Hill North Carolina USA

[9] current address: Roche Pharma Research and Early Development, Roche Innovation Center Basel, F. Hoffmann-La Roche AG, 4070 Basel, Switzerland

[#] Current address: Department of Science and Technology – Advanced Science and Technology Institute, UP Technology Park Complex, C.P. Garcia Ave., Diliman, Quezon City, Philippines

[##] Current address: Department of Chemistry, Eastern Visayas State University, Tacloban City, Philippines

[*]Corresponding authors. Email: ccaffrey@ucsd.edu and kbeis@imperial.ac.uk

[†]These authors contributed equally

Document contents:

Materials and Methods: pS4-S10
Figure S1: p S11
Figure S2: p S12
Figure S3: p S13-S16
Figure S4: p S17
Figure S5: p S18
Table S1: p S19
Table S2: p S20
Table S3: p S21
Supplementary References: p S22-S23

Materials and Methods

No unexpected or unusually high safety hazards were encountered.

Cloning of the kinase domain of SmVKR

The full-length native sequence of the *S. mansoni* VKR gene was synthesized. The constructs were codon optimized for expression in insect cells. All constructs were cloned into the pOPINF vector using infusion cloning [1]. The use of the pTriEx2 vector suite for construction of the pOPIN series to express protein from multiple hosts has been described previously [1]. The construct has an N-terminal hexa-histidine tag with rhinovirus 3C protease cleavage sites. The PCR reaction was carried out using KOD Hi-Fi polymerase according to manufacture recommendations (Novagen). The PCR products were purified using AMPure XP magnetic beads and the infusion reactions carried out using the linearized pOPINF based on our published protocol [1]. Construct verification was done using PCR with specific forward and reverse primers, and by DNA sequencing.

Baculovirus construction and expression in insect cells

Sf9 cells were seeded at 0.5x 10^6 cells/mL in a 24-well deep-well block (1.5 mL each well) and grown overnight to ~1x 10^6 cells/mL. For virus construction, 100 ng of the pOPINF_VKR2 baculovirus transfer plasmids (1 μg/μL) were mixed with 250 ng Bsu36I linearized DsRed bacmid (0.1 μg/μL) and 1.5 uL FuGENE HD Transfection Agent (Promega) in 50 μL Sf-900 II SFM. The transfection cocktail was thoroughly mixed, incubated for 30 minutes at RT, and then gently added to the Sf9 cells. The cultures were incubated for one week at 27°C and the culture medium (P0 virus stock) harvested. For P1 generation, 10 μL of the P0 stock (1:300 dilutions) was used to infect SF9 cells at 1x 10^6 cells/mL. After one week, supernatant (P1

stock) was collected and used to produce a P2 virus stock by infecting 50 mL of SF9II cells with 0.5 mL P1 stock. The resulting supernatant was sterile filtered, and stored at 4 C.

Protein expression was tested at small-scale by adding 3 or 30 uL of the P1 $SmVKR2_{KD}$ baculovirus stocks to 3 mL of Sf9 cells at $1x10^{6}$ cells/mL in each well grown in 24 deep, round-bottom well plates. Cells infected with a baculovirus expressing GFP were included as a control. After incubating the cells for 48 and 72 h at 27°C, soluble expression of the kinase domain was determined using small-scale Ni-NTA purification followed by SDS-PAGE and western blotting. Briefly, cell pellets were pelleted by centrifugation for 15 min at 6000 x g and then resuspended in lysis buffer (50 mM Tris, pH 7.5, 500 mM NaCl, 30 mM imidazole, 0.2 % Tween) supplemented with protease inhibitors and Benzonase nuclease. Following incubation for 30 min at - 80 °C, the His-tagged proteins were purified from the lysate using NiNTA magnet beads (Qiagen) according to the manufacturers protocol.

Constructs that showed good expression profiles were selected for large scale (2.5 L) expression and purification. Cells were infected with P2 virus using the same ratio by volume of cell culture: virus stock and time of harvest used in the small-scale expression test. Cells were harvested by centrifugation at 6000 x g for 15 min and disrupted by passing through a cell disruptor at 35.5 Kpsi. Cell lysates were then centrifuged at 30,000 x g for 30 min at 4°C. The cleared lysate was decanted from the insoluble fraction and filtered through a 0.22 μm bottle top filter before being applied to a 5 ml HisTrap column. The column was washed with 20-30 column volumes (CV) of Ni wash buffer (50 mM Tris, pH 7.5, 500 mM NaCl, 30 mM imidazole) and bound protein eluted in 50 mM Tris, pH 7.5, 500 mM NaCl and 500 mM imidazole. The eluted protein was applied to a Superdex 200 10/300 column equilibrated with gel filtration buffer (20 mM Tris, pH 7.5, 200 mM NaCl, 1 mM TCEP) and fractions monitored by A280. Peak fractions were analysed by SDS-PAGE, pooled, and concentrated to 14-16

mg/mL (Fig. S2). The concentrated protein was flash frozen in liquid nitrogen and stored at -80°C.

SPR assay development and fragment screen

The purified $SmVKR2_{KD}$ was captured via the His tag on a Biacore Series S nickel-NTA sensor chip in capture buffer (10 mM HEPES pH 7.4, 150 mM NaCl, 50 μM EDTA, 0.05% Tween 20), to obtain capture levels 10,300 – 11,400 RU. Analysis temperature was set to 25 °C and the sample compartment to 25 °C to maintain stability of the receptor on the surface. To increase the stability of the receptor, the $SmVKR2_{KD}$ was crosslinked to the surface by a 7 min injection of 1:1 ratio of 400 mM 1-ethyl-3-(3-dimethylaminopropyl) carbodiimide hydrochloride (EDC) and 100 mM N-hydroxy succinimide (NHS) prior to the capture of the SmVKR2 kinase domain onto activated Ni^{2+} surface. To confirm receptor activity, binding of ATPγS measured by injecting the ligand over the surface at 30 μL/min for 30 s and allowing to dissociate for 60 s. ATPγS was injected in concentration series from 2.06 – 500 μM. To test the stability of the capture-coupled receptor, ATPγS was injected at 500 μM concentration at 60-minute intervals for 35 hours and additionally in concentration series from 2.06 – 500 μM at the beginning and end of the screen totalling 48 hours.

The GSK PKIS2 library of 645 fragments was screened using Biacore T200 at concentration 100 μM in a running buffer composed of 20 mM Tris-HCl pH 7.5, 150 mM NaCl, 5 mM $MgCl_2$, 1 mM TCEP, 0.05% Tween 20, 3% DMSO against the SmVKR2 kinase domain, two blank surfaces and one additional reference receptor at a flow rate 30 μL/min. Association was measured for 30 s and dissociation for 60 s. ATPγS was injected at 250 μM throughout the screen to determine receptor activity. To identify hits, sensorgrams were individually inspected against all three surfaces (the $SmVKR2_{KD}$, blank flowcell and an additional reference receptor)

(Fig. S3). Twenty-seven compounds were then selected as potential hits and screened in concentration series from 0.41 – 100 μM. Subsequently, twelve SmVKR2 kinase domain fragment hits were selected and screened in 3-fold concentration series with top concentrations ranging from 2 – 50 μM depending on compound affinity in duplicate. Flow rate was set to 30 μL/min. Association and dissociation was measured for 60 s and 60 – 180 s, respectively. All experiments were run at 25 °C.

SPR data analysis

SPR data were referenced against blank surface and blank injections of running buffer. Scrubber 2 software (BioLogic Software) was used to process and analyse the data. For steady state affinity values, data were fitted to a 1:1 equilibrium model.

SmVKR2 kinase domain crystallization

Prior to crystallization experiments, the $SmVKR2_{KD}$ protein, 15 mg/ml, was incubated with 10 mM ATPγS and 5 mM $MgCl_2$ for 30 min on ice. Crystallization conditions were identified by screening the $SmVKR2_{KD}$ against the commercial screens Index, JBScreen Kinase, JCSG Plus and PACT using the sitting-drop vapour diffusion method. Plate-like crystals were grown from a solution containing 0.8 M succinic acid, pH 7.0 (Index screen) at 277 K after 1 month. The crystals diffracted X-rays to around 8 Å resolution. The crystal quality was further improved by the addition of 100 mM KCl and 50 mM sodium fluoride. Crystals were cryoprotected by the addition of 20% v/v glycerol in the mother liquor. Crystals were flash frozen in liquid nitrogen for data collection.

Data collection

Diffraction data from the SmVKR2$_{KD}$ at 3.0 Å were collected on I24 at Diamond Light Source at a wavelength of 0.92 Å using a Pilatus3 6M detector and processed using autoPROC [2]. Further processing was performed using the CCP4 suite [3]. The resolution of the data and anisotropy analysis were evaluated by the half-dataset correlation coefficient in Aimless (cut-off less than 0.5) [4]. The space group was determined to be *C2* with three copies of the SmVKR2$_{KD}$ in the asymmetric unit. The data collection statistics are summarized in Table S3.

Structure determination and refinement

The structure of the SmVKR2$_{KD}$ was determined by molecular replacement in Phaser [5] using the insulin-like growth factor 1-receptor kinase domain (PDB ID: 2OJ9) as a search model. Refinement to 3.0 Å was carried out in PHENIX [6]. After rigid body and restrained refinement, extra electron density corresponding to ADP molecules was identified. ADP was only built and refined in two of the three copies as the density was weak in the third copy. The activation loop was partially modelled due to the density between residues 1124-1141 being too weak/disordered to confidently add side chains. All model rebuilding was performed in Coot [7].

The final model has an R_{work} of 22.1 % and an R_{free} of 27.8 %. The SmVKR2$_{KD}$ structure has 91.5 % of the residues in the favoured Ramachandran region with one outlier, S1199, as calculated by MolProbity [8]. The refinement statistics are summarized in Table S3.

***In silico* docking**

The molecular docking of ATP, ADP, ATPγS and the inhibitors was performed with AutoDock Vina (v4.2.6) [9]. The receptor and ligands were prepared using AutoDock Tools (v1.5.7). The

search box was centred around the ATP binding site and the grid box size was set to 18 x 12 x 22 $Å^3$ in the dimensions of *x*, *y* and *z* using a 1.0 Å spacing. The pose of the docked ATP and ADP were compared against the crystal structure to provide us with confidence for the poses of the inhibitors. The top ranked poses were analysed in ChimeraX [10].

Preparation of S. *mansoni*

The acquisition, preparation and *in vitro* maintenance of *S. mansoni* have been described [11], [12]. We use a Naval Medical Research Institute (NMRI) isolate of *S. mansoni* that is cycled between *Biomphalaria glabrata* snails and male Golden Syrian hamsters (infected at 4-6 weeks of age) as intermediate and definitive hosts, respectively. Briefly, adult worms were harvested from hamsters 42 days post-infection in RPMI or DMEM, and washed five times prior to maintenance overnight at 37 °C and 5% CO_2 in Basch medium [13] containing 4% heat-inactivated FBS, 100 µg/ml streptomycin and 100 U/ml penicillin.

Ex vivo* activity against adult *S. mansoni

Ex vivo screens with adults employed 24-well flat-bottomed plates and approximately five males, two to three of which were paired to females. Screens incorporated a final compound concentration of 10 µM in DMSO (0.5% final concentration). The complex and often dynamic phenotypic responses that the schistosome parasite is capable of were observed using a Zeiss Axiovert A1 inverted microscope at 2, 5 and 24 h. Observations were classified using a constrained nomenclature that involves simple "descriptors" to convey changes in shape, motility and appearance, and the inability of the male worm to adhere to the bottom of the well, compared to DMSO controls [11], [14], [15]. Each descriptor was given a value of 1 and these were added up to yield a "severity score" with a maximum of 4. Evidence of degeneracy or death was awarded a value of 4 as was damage to the tegument (surface), on the understanding this would compromise survival *in vivo*. Scores were averaged across 3-5 individual experiments.

In addition to observational assessments, the video camera-based WormAssay was employed to measure average motility of the worms per well [16], [17].

Kinase activity assay

The ADP-Glo Kinase Assay (Promega) was used to determine the IC_{50} values of $SmVKR2_{KD}$. The assay was performed according to the manufacturer's recommendations without any modifications. The optimal concentration of the $SmVKR2_{KD}$ in the assay was established as 0.7 μM. The autophosphorylation activity was measured at an ATP concentration of 10 μM to aid the identification of competitive inhibitors as recommended by the ADP-Glo Kinase Assay (Promega) protocol. The autophosphorylation activity of the $SmVKR2_{KD}$ was measured in the presence and absence of inhibitors at various concentrations. The reaction was left at room temperature for 30 min prior to measuring ADP production using the ADP-Glo Kinase Assay kit (Promega). The luminescence was measured using a CLARIOstar plate reader. Data analysis and IC_{50} calculations were performed using GraphPad Prism.

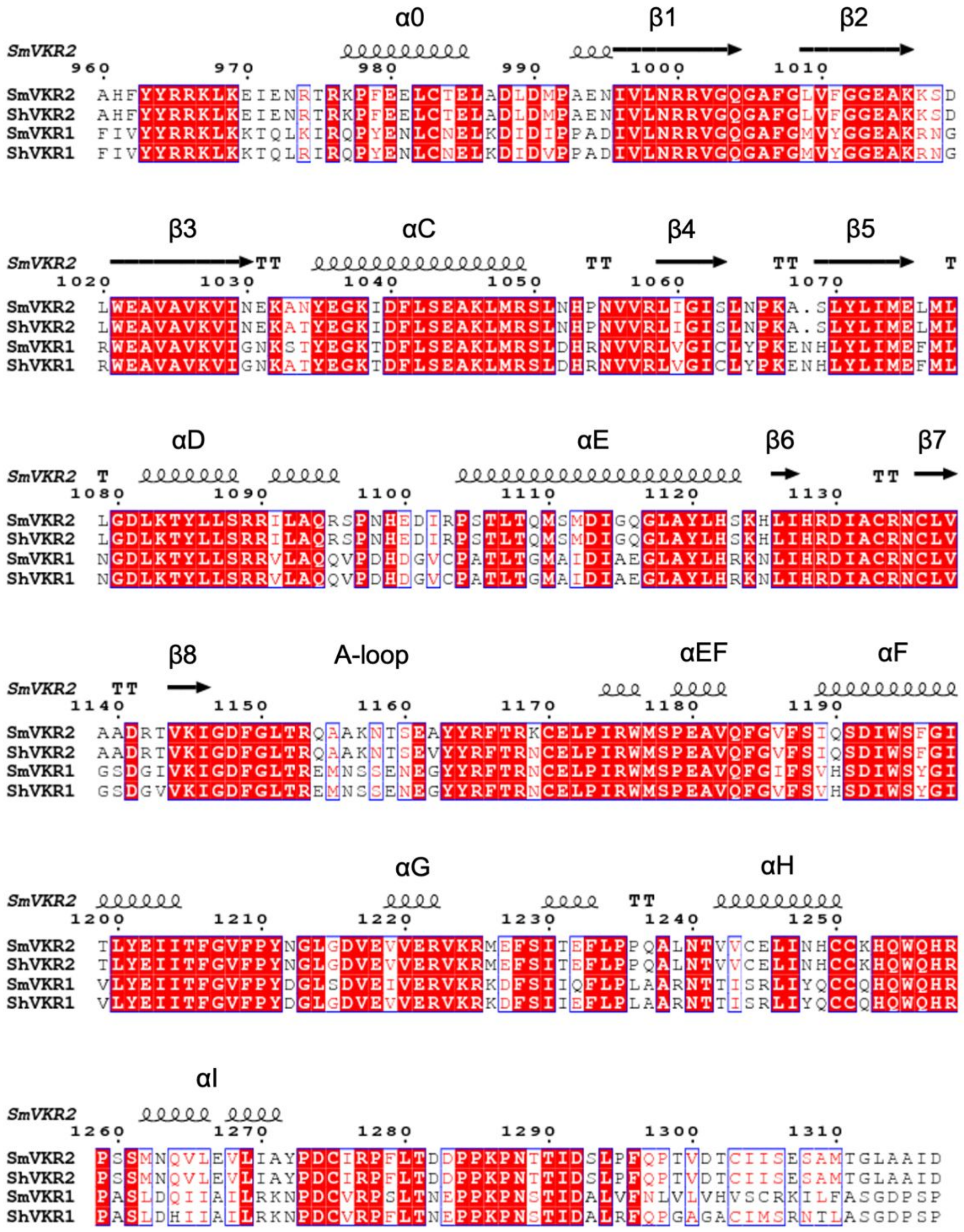

Figure S1. Sequence alignment of VKR1 and VKR2 kinase domains from *S. haematobium* and *S. mansoni* using Clustal Omega. Secondary structure elements are labelled as in Fig. 2.

A

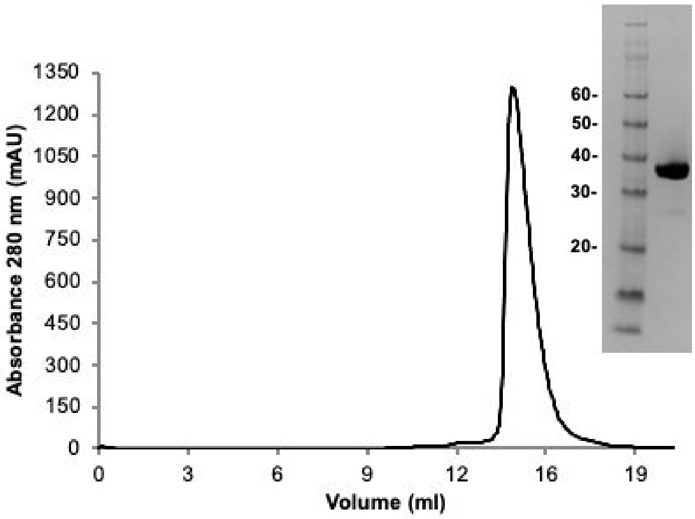

B

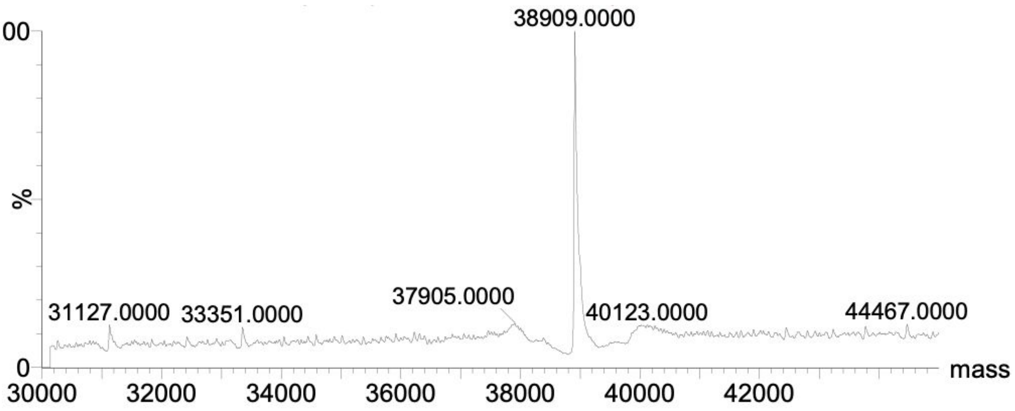

Figure S2. Purification of the SmVKR2$_{KD}$. (A) Size exclusion chromatography profile of the SmVKR2 kinase domain. The protein appears monodisperse. Inset corresponds to the Coomassie stained SDS-PAGE of the pulled fractions after the size exclusion chromatography. (B) Mass spectrometry analysis of the purified protein. The theoretical molecular weight of the construct is 38913 and the calculated 38909 suggests that the kinase has not been post-translationally modified during overexpression.

GSK1520489

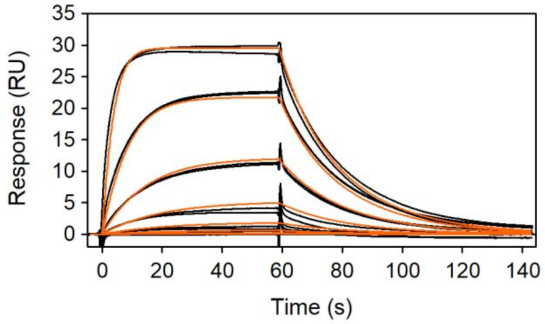

GSK986310

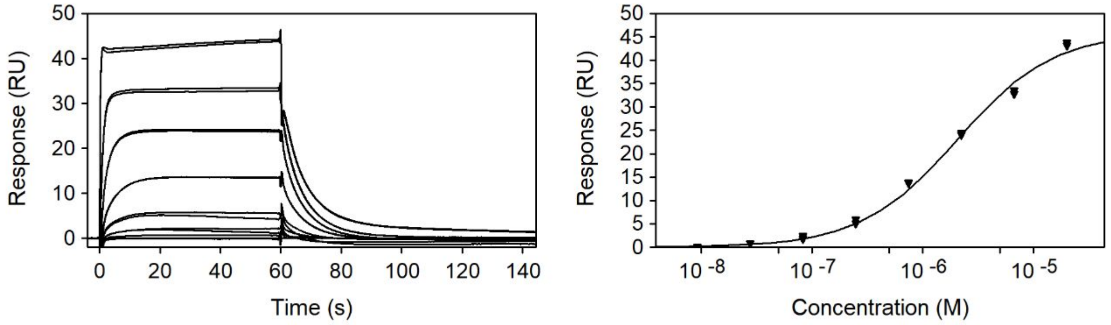

GSK1292139

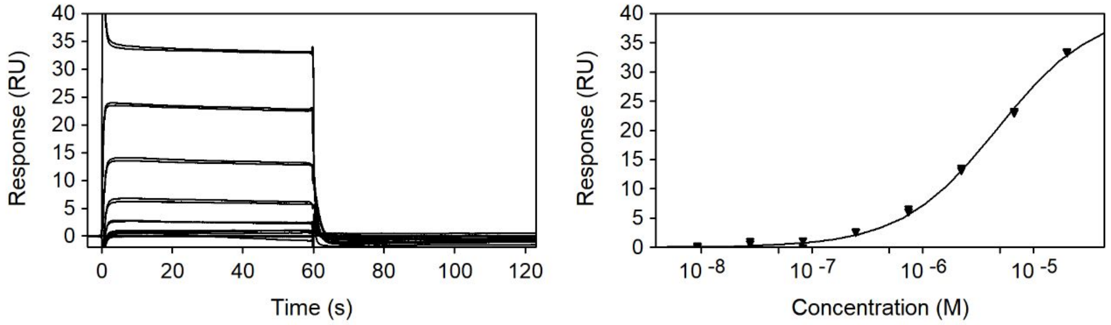

GW682569

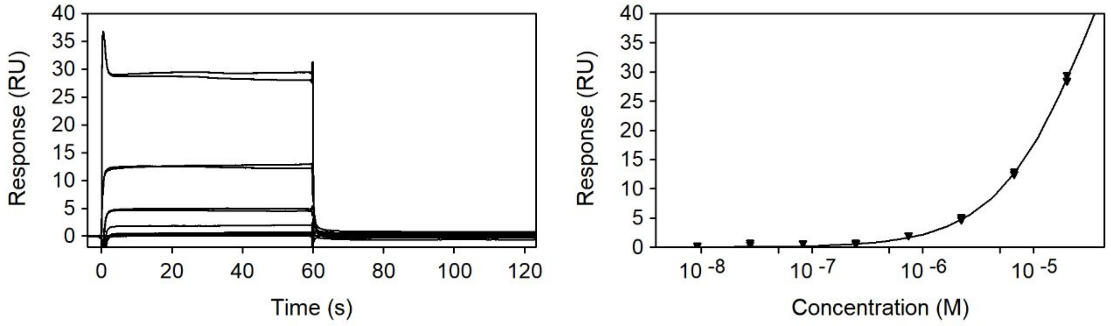

GSK993273

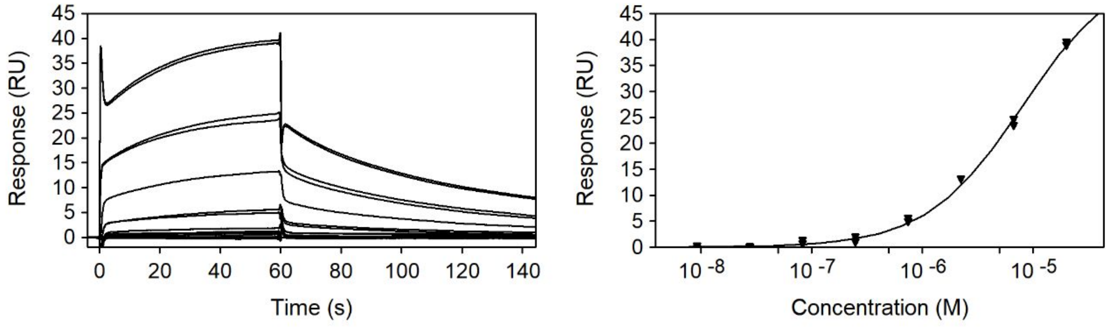

GSK977620

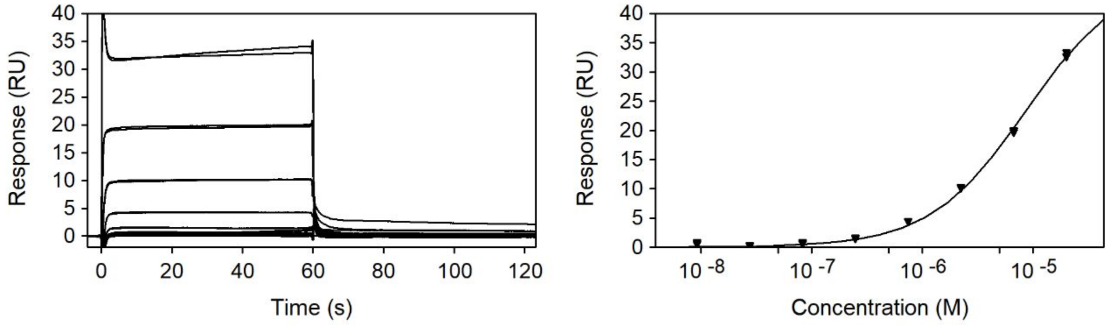

GSK977617

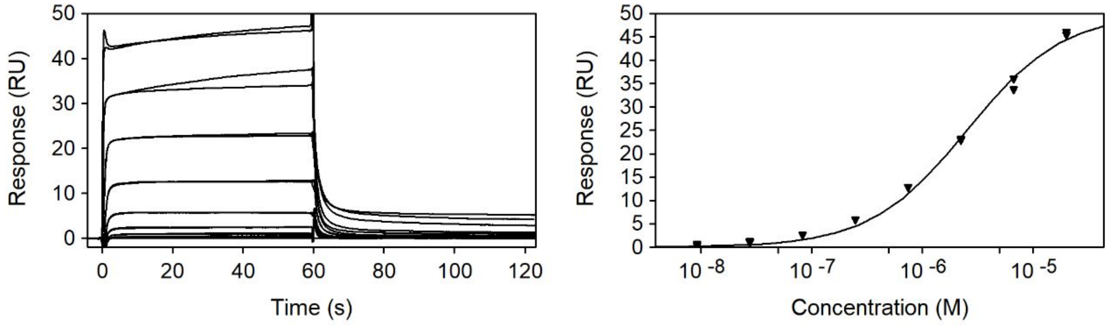

SB-642124

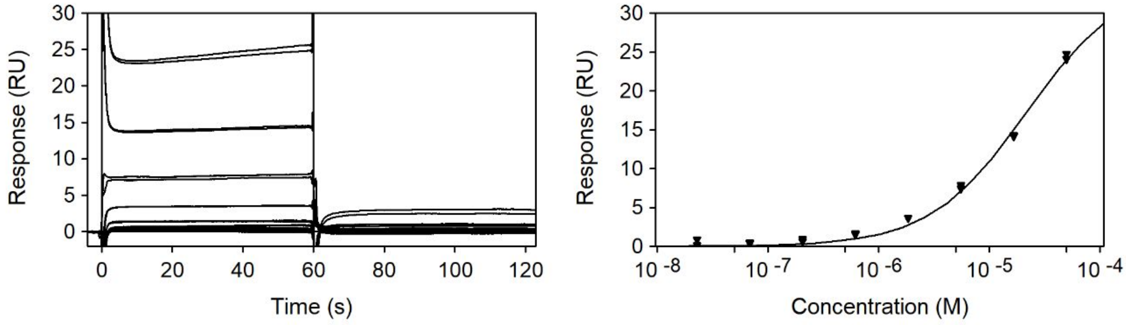

SB-710363

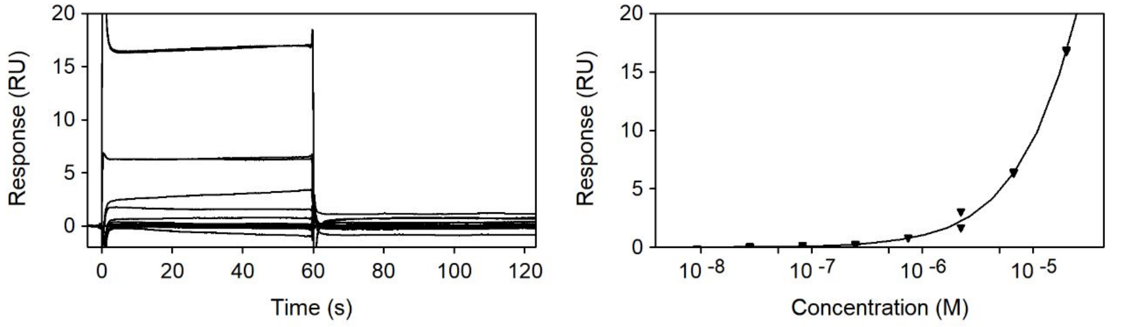

SKF-12778

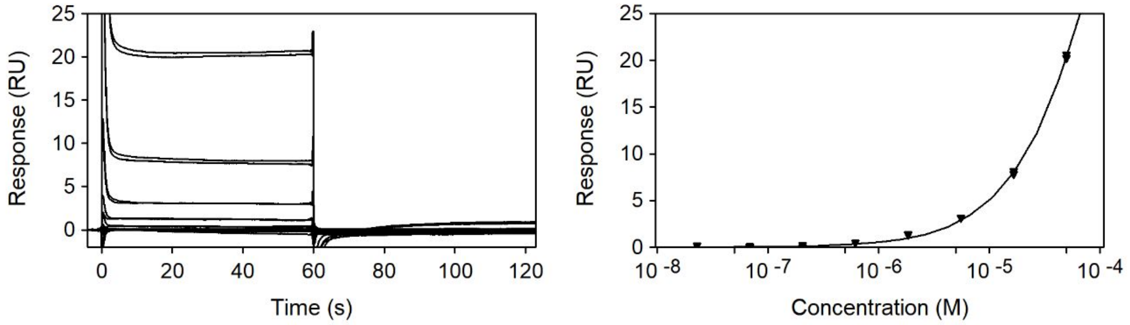

GW789449

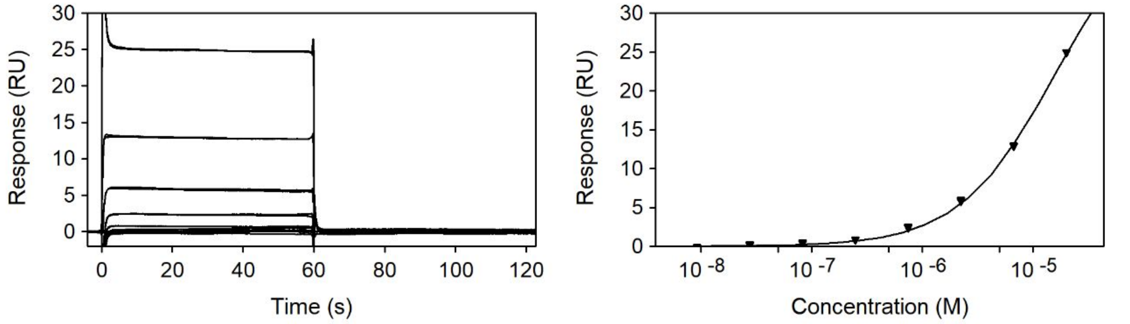

GW696155

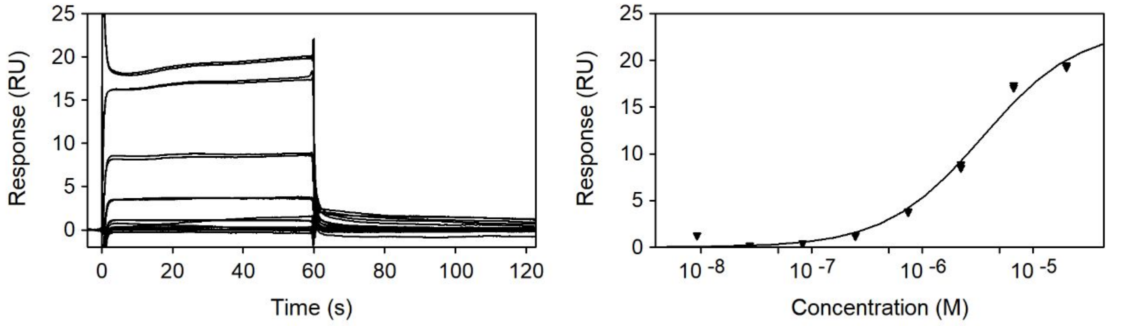

Figure S3. SPR sensorgrams for compounds binding to the immobilised $SmVKR2_{KD}$. Each compound was injected in duplicate at concentration series 2 – 50 μM. Data were fit using

steady state affinity model except compound GSK1520489 where 1:1 binding kinetic fit was used to determine binding affinity. Red lines represent the fit. Compound GSK993273 presents a partial complex binding and therefore the affinity value is approximate.

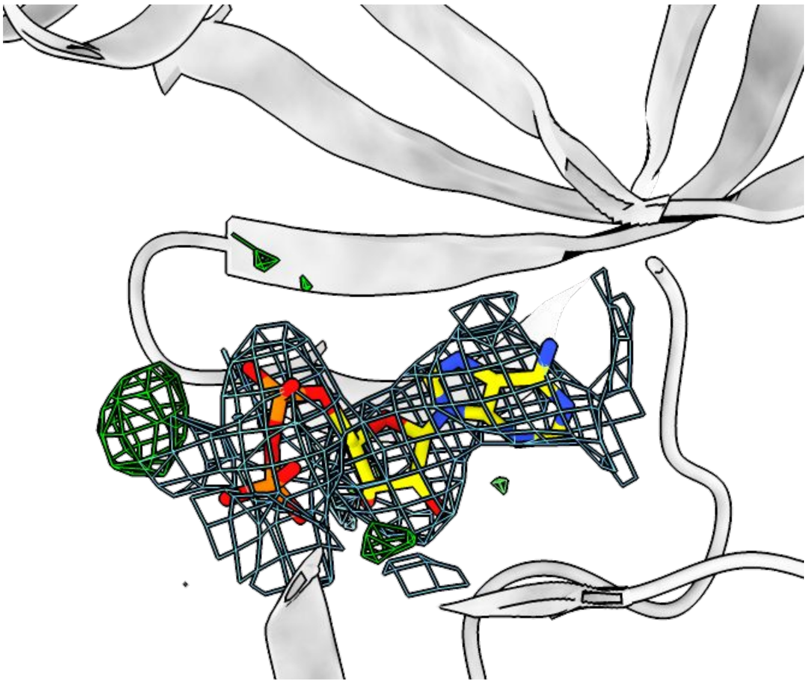

Figure S4. Electron density for ADP. 2Fo-Fc electron density map around the ADP molecule for the refined structure of $SmVKR2_{KD}$ (contoured at 1.0 σ, blue mesh). Fo-Fc electron density can be observed next to the ADP that could correspond to the cleaved thiophosphate group (contoured at 3.0 σ, green mesh). The maps have been cropped at a 5 Å radius around the ADP molecule. ADP is shown in sticks and the $SmVKR2_{KD}$ as cartoons.

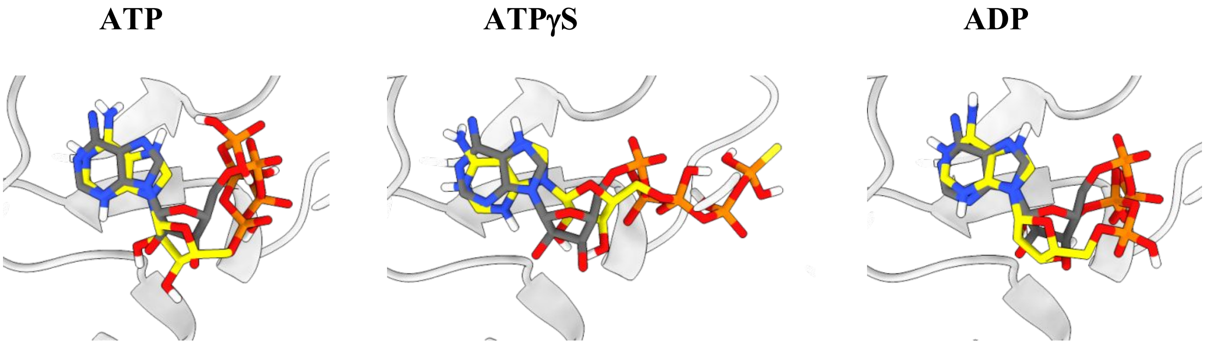

Figure S5. Docking of nucleotides in the SmVKR2$_{KD}$ active site. The docked ATP, ATPγS and ADP adopt a similar binding pose to the ADP-bound crystal structure (left, middle right panel, respectively); small deviations are observed which are acceptable within the limits of the docking. The nucleotides are shown in sticks; the crystallographic carbon of ADP is in dark grey and the docked nucleotides in yellow. The SmVKR2$_{KD}$ is shown as a grey cartoon.

Table S1. Several of the identified compounds by SPR lacked bioactivity. Descriptors and severity scores as in Table 2. Data were derived from 3-5 biological replicates each performed as a singleton.

	Descriptors			Severity scores		
**Compound**	**2 h**	**5 h**	**24 h**	**2 h**	**5 h**	**24 h**
**GSK977617**	none	none	none	0	0	0
**GSK1292139**	none	none	none	0	0	0
**GSK993273**	none	none	none	0	0	0
**GSK977620**	none	none	none	0	0	0
**GW789449**	none	none	none	0	0	0
**GW682569**	none	none	none	0	0	0
**SB-642124**	none	none	none	0	0	0
**SKF-12778**	none	none	none	0	0	0

Table S2. Maximum Common Substructure (MCS) as calculated by the Tanimoto coefficient for the GSK1520489, GSK986310, GW696155 and SB-710363 compounds that displayed *ex vivo* anti-schistosomal activity. The Tanimoto coefficient has a range from 0 to 1 with higher values indicating greater similarity than lower ones.

	GSK1520489	GSK986310	GW696155	SB-710363
**GSK1520489**	1			
**GSK986310**	0.37	1		
**GW696155**	0.24	0.20	1	
**SB-710363**	0.17	0.20	0.22	1

Chemical structures of the compounds analysed:

GSK1520489

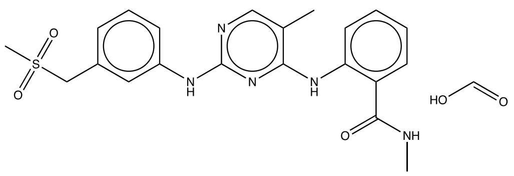

GSK986310

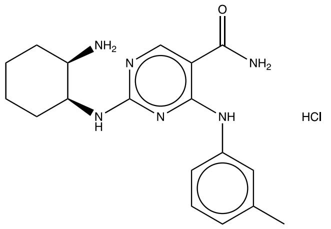

GW696155

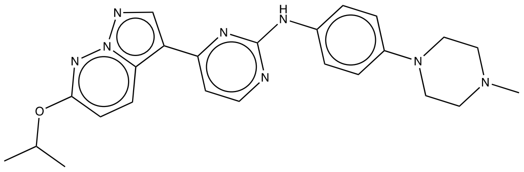

SB-710363

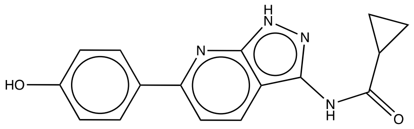

Table S3. Data collection and refinement statistics. Values in parentheses refer to data in the highest resolution shell.

**Data collection statistics**	**$SmVKR2_{KD}$**
Beamline	I24
Space group	C 1 2 1
Resolution (Å)	102.24-3.06 (3.29-3.06)
Cell Dimensions (Å)	
a, *b*, *c* (Å)	206.25 97.23 72.94
α, β, γ (°)	90.0, 97.5, 90.0
Number of reflections	39251 (2026)
Number of unique reflections	16396 (821)
Completeness (%) *spherical*#	60.8 (15.9)
Completeness (%) *ellipsoidal*#	87.3 (53.8)
Multiplicity	4.6 (5.8)
R_{merge}	0.084 (0.82)
Mean(I)/σ(I)	2.4 (2.5)
CC(1/2)	0.99 (0.42)

**Refinement statistics**	
R_{work} (%)	21.7
R_{free} (%)	25.4

**Rmsd from ideal values**	
Bonds (Å)	0.004
Angle (°)	0.82
Ramachandran favored (%)	91.52
Ramachandran allowed (%)	8.37
Ramachandran plot outlier (%)	0.11

a “spherical” looks at all data within a specific, spherical resolution range/bin, so this is the usual, well known way of looking at data as a function of resolution. “Ellipsoidal” additionally requires that a data point be within the fitted ellipsoid in order to be considered.

Supplementary References

1. Berrow, N. S.; Alderton, D.; Sainsbury, S.; Nettleship, J.; Assenberg, R.; Rahman, N.; Stuart, D. I.; Owens, R. J., A versatile ligation-independent cloning method suitable for high-throughput expression screening applications. *Nucleic Acids Res* **2007,** *35* (6), e45.
2. Vonrhein, C.; Flensburg, C.; Keller, P.; Sharff, A.; Smart, O.; Paciorek, W.; Womack, T.; Bricogne, G., Data processing and analysis with the autoPROC toolbox. *Acta Crystallogr D Biol Crystallogr* **2011,** *67* (Pt 4), 293-302.
3. Collaborative Computational Project, N., The CCP4 Suite: Programs for Protein Crystallography. *Acta Crystallogr D Biol Crystallogr* **1994,** *50*, 760-763.
4. Evans, P., Scaling and assessment of data quality. *Acta crystallographica. Section D, Biological crystallography* **2006,** *62* (Pt 1), 72-82.
5. McCoy, A. J.; Grosse-Kunstleve, R. W.; Adams, P. D.; Winn, M. D.; Storoni, L. C.; Read, R. J., Phaser crystallographic software. *J. Appl. Cryst.* **2007,** *40*, 658-674.
6. Terwilliger, T. C.; Grosse-Kunstleve, R. W.; Afonine, P. V.; Moriarty, N. W.; Zwart, P. H.; Hung, L. W.; Read, R. J.; Adams, P. D., Iterative model building, structure refinement and density modification with the PHENIX AutoBuild wizard. *Acta crystallographica. Section D, Biological crystallography* **2008,** *64* (Pt 1), 61-9.
7. Emsley, P.; Cowtan, K., Coot: model-building tools for molecular graphics. *Acta Crystallogr D Biol Crystallogr* **2004,** *60* (Pt 12 Pt 1), 2126-32.
8. Chen, V. B.; Arendall, W. B., 3rd; Headd, J. J.; Keedy, D. A.; Immormino, R. M.; Kapral, G. J.; Murray, L. W.; Richardson, J. S.; Richardson, D. C., MolProbity: all-atom structure validation for macromolecular crystallography. *Acta crystallographica. Section D, Biological crystallography* **2010,** *66* (Pt 1), 12-21.
9. Trott, O.; Olson, A. J., AutoDock Vina: improving the speed and accuracy of docking with a new scoring function, efficient optimization, and multithreading. *J Comput Chem* **2010,** *31* (2), 455-61.
10. Pettersen, E. F.; Goddard, T. D.; Huang, C. C.; Meng, E. C.; Couch, G. S.; Croll, T. I.; Morris, J. H.; Ferrin, T. E., UCSF ChimeraX: Structure visualization for researchers, educators, and developers. *Protein Sci* **2021,** *30* (1), 70-82.
11. Abdulla, M. H.; Ruelas, D. S.; Wolff, B.; Snedecor, J.; Lim, K. C.; Xu, F.; Renslo, A. R.; Williams, J.; McKerrow, J. H.; Caffrey, C. R., Drug discovery for schistosomiasis: hit and lead compounds identified in a library of known drugs by medium-throughput phenotypic screening. *PLoS Negl Trop Dis* **2009,** *3* (7), e478.
12. Abdulla, M. H.; Lim, K. C.; Sajid, M.; McKerrow, J. H.; Caffrey, C. R., Schistosomiasis mansoni: novel chemotherapy using a cysteine protease inhibitor. *PLoS Med* **2007,** *4* (1), e14.
13. Basch, P. F., Cultivation of Schistosoma mansoni in vitro. I. Establishment of cultures from cercariae and development until pairing. *J Parasitol* **1981,** *67* (2), 179-85.
14. Probst, A.; Nguyen, T. N.; El-Sakkary, N.; Skinner, D.; Suzuki, B. M.; Buckner, F. S.; Gelb, M. H.; Caffrey, C. R.; Debnath, A., Bioactivity of Farnesyltransferase Inhibitors Against Entamoeba histolytica and Schistosoma mansoni. *Front Cell Infect Microbiol* **2019,** *9*, 180.
15. Long, T.; Rojo-Arreola, L.; Shi, D.; El-Sakkary, N.; Jarnagin, K.; Rock, F.; Meewan, M.; Rascon, A. A., Jr.; Lin, L.; Cunningham, K. A.; Lemieux, G. A.; Podust, L.; Abagyan, R.; Ashrafi, K.; McKerrow, J. H.; Caffrey, C. R., Phenotypic, chemical and functional

characterization of cyclic nucleotide phosphodiesterase 4 (PDE4) as a potential anthelmintic drug target. *PLoS Negl Trop Dis* **2017,** *11* (7), e0005680.
16. Marcellino, C.; Gut, J.; Lim, K. C.; Singh, R.; McKerrow, J.; Sakanari, J., WormAssay: a novel computer application for whole-plate motion-based screening of macroscopic parasites. *PLoS Negl Trop Dis* **2012,** *6* (1), e1494.
17. Bibo-Verdugo, B.; Wang, S. C.; Almaliti, J.; Ta, A. P.; Jiang, Z.; Wong, D. A.; Lietz, C. B.; Suzuki, B. M.; El-Sakkary, N.; Hook, V.; Salvesen, G. S.; Gerwick, W. H.; Caffrey, C. R.; O'Donoghue, A. J., The Proteasome as a Drug Target in the Metazoan Pathogen, Schistosoma mansoni. *ACS Infect Dis* **2019,** *5* (10), 1802-1812.
